# Investigation of Fe_3_O_4_@boehmite NPs as efficient and magnetically recoverable nanocatalyst in the homoselective synthesis of tetrazoles†

**DOI:** 10.1039/d2ra04759d

**Published:** 2022-11-22

**Authors:** Parisa Moradi

**Affiliations:** Department of Chemistry, Faculty of Science, Ilam University P. O. Box 69315516 Ilam Iran parisam28@yahoo.com p.moradi@ilam.ac.ir +98 841 2227022 +98 841 2227022

## Abstract

Magnetic boehmite nanoparticles (Fe_3_O_4_@boehmite NPs) were synthesized from a hybrid of boehmite and Fe_3_O_4_ nanoparticles. At first, boehmite nanoparticles (aluminum oxide hydroxide) were prepared *via* a simple procedure in water using commercially available materials such as sodium hydroxide and aluminum nitrate. Then, these nanoparticles were magnetized using Fe_3_O_4_ NPs in a basic solution of FeCl_2_·4H_2_O and FeCl_3_·6H_2_O. Fe_3_O_4_@boehmite NPs have advantages of both boehmite nanoparticles and Fe_3_O_4_ magnetic materials. Magnetic boehmite nanoparticles have been characterized by various techniques such as TEM, SEM, EDS, WDX, ICP, FT-IR, Raman, XRD and VSM. SEM and TEM images confirmed that particles size are less than 50 nm in diameter with a cubic orthorhombic structure. Then, Fe_3_O_4_@boehmite NPs were applied as a homoselective, highly efficient, cheap, biocompatibility, heterogeneous and magnetically recoverable nanocatalyst in the synthesis of 5-substituted 1*H*-tetrazole derivatives. Fe_3_O_4_@boehmite NPs can be recycled for several runs in the synthesis of tetrazoles. Also, all tetrazoles were isolated in high yields, which reveals high activity of Fe_3_O_4_@boehmite NPs in the synthesis of tetrazole derivatives. Fe_3_O_4_@boehmite NPs shows a good homoselectivity in synthesis of 5-substituted 1*H*-tetrazole derivatives.

## Abstract 2

Magnetic boehmite nanoparticles were synthesized from a hybrid of boehmite and Fe_3_O_4_ nanoparticles. Then, it was applied as a homoselective, highly efficient, cheep, heterogeneous and recoverable nanocatalyst in the synthesis of tetrazole derivative.

## Graphical abstract

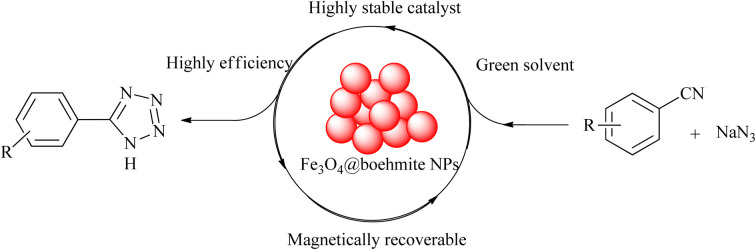

## 1 Introduction

**Fig. 1:**
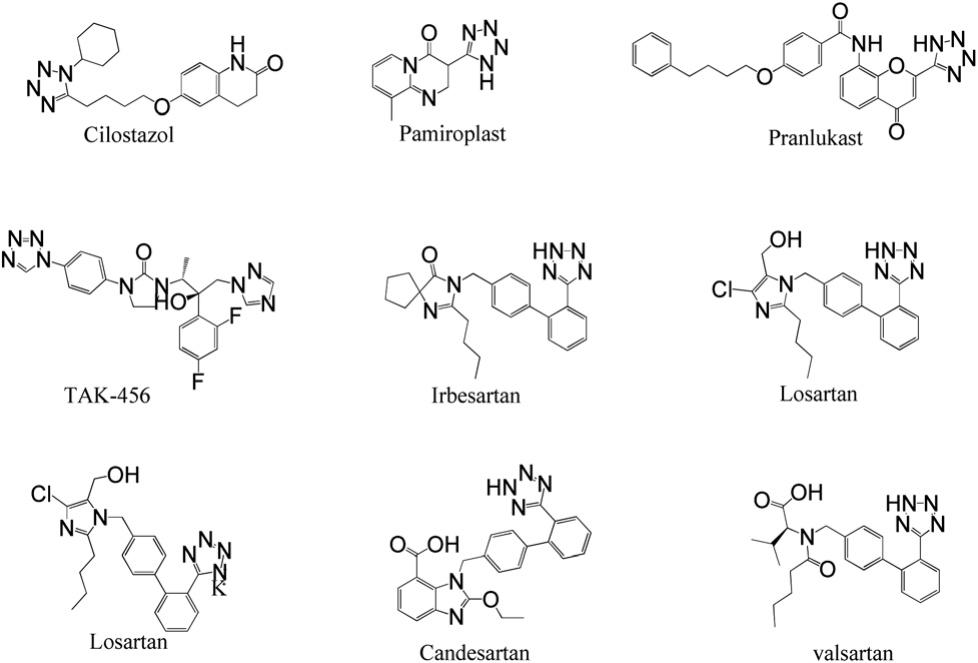
Several pharmacologically compounds of tetrazoles.

In homogeneous catalyst systems, good catalytic activity is usually observed, due to the solubility of the catalyst in the reaction mixture and thus the easy access of the reactants to the catalyst sites. Therefore homogeneous catalysts perform better practicality than heterogeneous catalysts.^1–3^ However, in the homogeneous catalyst systems, purification of the products, separation and recovery of the catalyst is often difficult, costly and time consuming. Therefore, this drawbacks have limited the use of the homogeneous catalysts despite high activity and selectivity.^4^ Also, clean technology and green chemistry require the use of heterogeneous and recyclable catalysts.^3–6^ While we need to catalytic systems which have high activity and recyclability. This goal is achieved through the use of nanocatalysts which are the bridge between homogeneous and heterogeneous catalysts.^7,8^ However, nanoparticles (NPs) are not fully recycled by conventional and inexpensive methods such as centrifugation or filtration due to their very small size. All of these problems can be overcome using magnetic nanocatalysts or stabilizing catalyst species on magnetic substrates.^8–10^ In the absence of an external magnetic field, the magnetic nanoparticles (MNPs) are dispersed in the reaction mixture and make available high surface to the reacting molecules. More important, at the end of the reaction, they are quickly, easily and completely recovered from the reaction mixture using an external magnet.^11–16^ But these MNPs are not very stable for long times.^17^ Therefore, many organic or inorganic covers have been used to increase the MNPs stability.^18–21^ One of the most valuable and cheapest mineral compounds which is rarely used as MNPs cover is boehmite nanoparticles (BNPs) which are synthesized using inexpensive and available materials and very simple method in the aqueous environment.^22^ Boehmite is actually one of the polymorphs phases of aluminum oxide which called aluminum oxyhydroxide (AlOOH).^23–27^ BNPs with high surface area have high hydroxyl groups on their surface that causes it to be used as a coating.^28,29^ Therefore, magnetic boehmite nanoparticles (Fe_3_O_4_@boehmite NPs) have advantages of homogeneous catalyst (such as large surface area and activity), high stability (such as boehmite NPs) and MNPs (such as easily and magnetically separation using an external magnet). For this purpose, in this project, Fe_3_O_4_@boehmite NPs were synthesized and used as a highly efficient, environmentally friendly and magnetically reusable nanocatalyst in the synthesis of 5-substituted 1*H*-tetrazoles. The procedure synthesis of Fe_3_O_4_@boehmite NPs is cheap, simple and environmentally friendly. 5-Substituted tetrazoles were used in drugs and they are used as herbicides, anti-HIV drug candidates, analgesics, antimicrobial, anti-proliferative, anti-inflammatory, and anticancer agents.^26,30–38^ For example, Candesartan, Valsartan, Irbesartan, Losartan, Cilostazol, TAK-456, Pemiroplast and Pranlukast (Fig. 1) are several pharmacologically important of tetrazoles.^39–41^

## 2 Experimental

### 2.1 Preparation of Fe_3_O_4_@boehmite NPs catalyst

The BNPs were synthesized according to previously reported procedure.^44^ Then, the obtained BNPs (2 g) dispersed in water at 80 °C, and then FeCl_2_·4H_2_O (7.5 mmol, 1.49 g) and FeCl_3_·6H_2_O (11.5 mmol, 3.1 g) added to the mixture. The reaction mixture stirred under N_2_ atmosphere. Under continuous stirring, NaOH (50 mL, 10%) added into the reaction mixture. The obtained mixture stirred for 2 h at 90 °C which black Fe_3_O_4_@boehmite NPs prepared. The obtained Fe_3_O_4_@boehmite NPs washed with distilled water and separated by external magnet each time.

### 2.2 General procedure for synthesis of tetrazoles catalyzed by Fe_3_O_4_@boehmite NPs

A mixture of sodium azide (1.5 mmol) and benzonitrile derivative (1 mmol) stirred in the presence of Fe_3_O_4_@boehmite NPs catalyst (0.015 g) in PEG at 120 °C. Reaction times controlled by TLC. After completion of the reaction, the Fe_3_O_4_@boehmite NPs catalyst isolated using an external magnet and the tetrazole products extracted by ethyl acetate and aqueous solution of HCl (4 N). The organic solvent dried over anhydrous Na_2_SO_4_, and evaporated to give the tetrazole products.

### 2.3 Selected spectral data

#### 2.3.1 2-(1*H*-Tetrazol-5-yl)benzonitrile

^1^H NMR (400 MHz, CDCl_3_): *δ*_H_ = 8.11–8.06 (t, *J* = 8 Hz, 2H), 7.96–7.90 (t, *J* = 12 Hz, 1H), 7.81–7.75 (t, *J* = 20 Hz, 1H) ppm.

#### 2.3.2 5-(3-Nitrophenyl)-1*H*-tetrazole

^1^H NMR (400 MHz, CDCl_3_): *δ*_H_ = 14.96 (br, 1H), 8.84–8.83 (t, *J* = 4 Hz, 1H), 8.49–8.39 (m, 2H), 7.93–7.88 (t, *J* = 8 Hz, 1H) ppm.

#### 2.3.3 5-(4-Bromophenyl)-1*H*-tetrazole

^1^H NMR (400 MHz, CDCl_3_): *δ*_H_ = 7.98–7.96 (d, *J* = 8 Hz, 2H), 7.84–7.82 (d, *J* = 8 Hz, 2H) ppm.

#### 2.3.4 5-(4-Nitrophenyl)-1*H*-tetrazole

^1^H NMR (400 MHz, CDCl_3_): *δ*_H_ = 14.76 (br, 1H), 8.45–8.41 (d, *J* = 12 Hz, 2H), 8.30–8.27 (d, *J* = 12 Hz, 2H) ppm.

## 3 Results and discussion

**Fig. 2:**
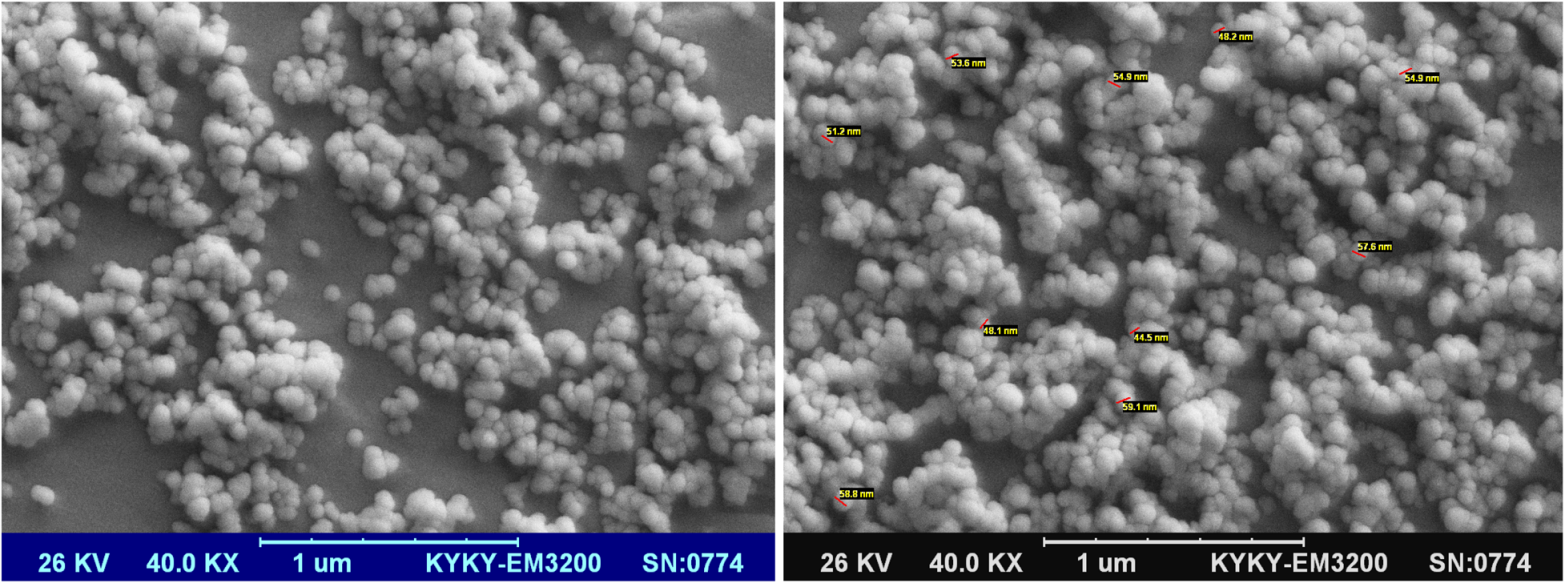
SEM images of Fe_3_O_4_@boehmite NPs.

In this project, to combine the advantages of both MNPs and boehmite nanoparticles, magnetic boehmite nanoparticles (Fe_3_O_4_@boehmite NPs) synthesized using Fe_3_O_4_ nanoparticles and available materials through environmentally friendly and very simple procedure in aqueous media. In the next step, Fe_3_O_4_@boehmite NPs characterized by transmission electron microscopy (TEM), scanning electron microscopy (SEM), wavelength dispersive X-ray spectroscopy (WDX), energy-dispersive X-ray spectroscopy (EDS), Inductively coupled plasma (ICP), Fourier transform infrared spectroscopy (FT-IR), Raman spectroscopy, X-ray diffraction (XRD) and vibrating-sample magnetometer (VSM) techniques. The scanning electron microscope was used to obtain high-resolution SEM images of Fe_3_O_4_@boehmite NPs. The SEM images were used to studying the morphology and diameter size of Fe_3_O_4_@boehmite NPs. SEM images of Fe_3_O_4_@boehmite NPs are show in Fig. 2 which indicate particles size of Fe_3_O_4_@boehmite NPs are less than 60 nm.

**Fig. 3:**
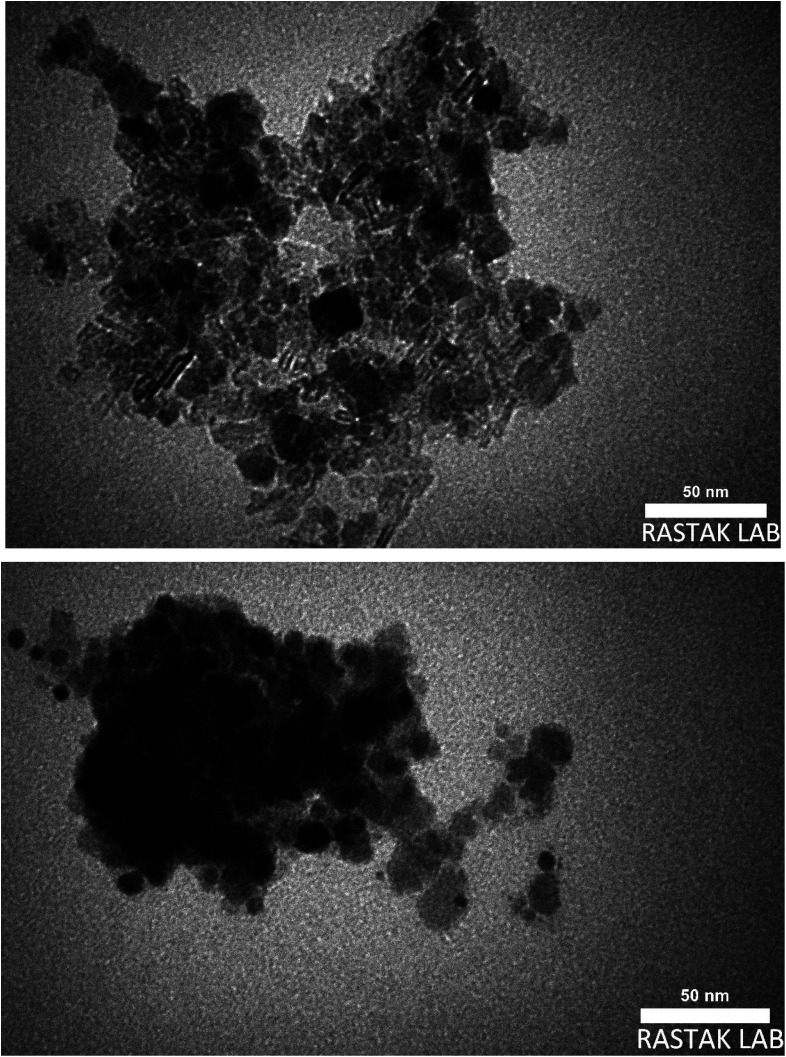
TEM images of Fe_3_O_4_@boehmite NPs.

Also, the TEM images were used to studying the morphology and size of Fe_3_O_4_@boehmite NPs. As shown in TEM images (Fig. 3), Fe_3_O_4_@boehmite NPs were formed in uniform shapes with quite homogeneous diameter.

**Fig. 4:**
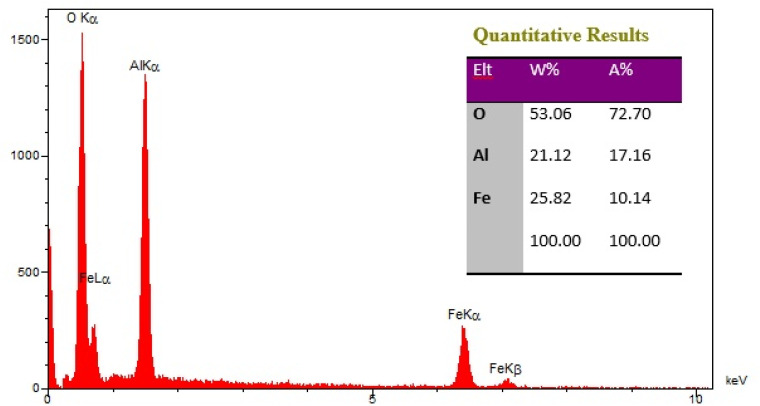
EDX spectrum of Fe_3_O_4_@boehmite NPs.

**Fig. 5:**
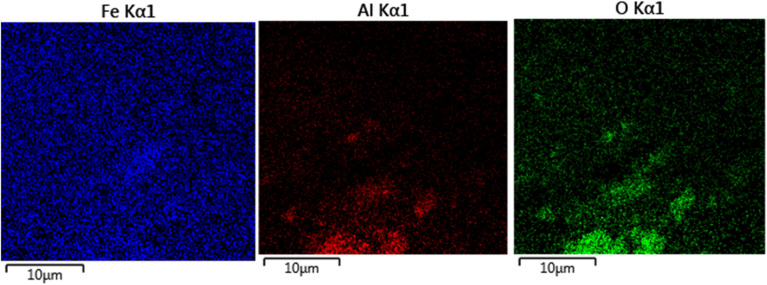
Elemental mapping of iron, aluminum and oxygen for Fe_3_O_4_@boehmite NPs.

The elements content of Fe_3_O_4_@boehmite NPs was studied by EDS analysis. The EDS analysis of Fe_3_O_4_@boehmite NPs is shown in Fig. 4 which indicate the presence of oxygen, iron and aluminum species. Also, the elements distribution of Fe_3_O_4_@boehmite NPs was studied by WDX analysis. The WDX analysis of Fe_3_O_4_@boehmite NPs is shown in Fig. 5 which indicate the uniform distribution of the elements in Fe_3_O_4_@boehmite NPs. As shown in the EDS diagram, no elements except oxygen, iron and aluminum elements were seen.

The exact amount of iron and aluminum elements in Fe_3_O_4_@boehmite NPs was obtained by ICP analysis, which found to be 5.7 × 10^−3^ mol g^−1^ and 3.5 × 10^−3^ mol g^−1^ respectively.

**Fig. 6:**
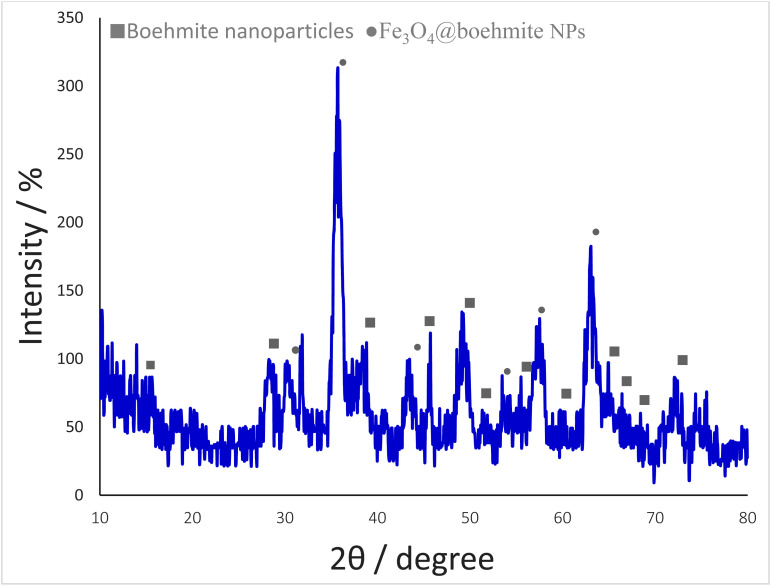
Normal XRD pattern of Fe_3_O_4_@boehmite NPs.

The normal XRD pattern of Fe_3_O_4_@boehmite NPs is shown in Fig. 6. The XRD pattern of Fe_3_O_4_@boehmite NPs shows the several peaks of 2*θ* values at 30.5° (220), 35.7° (311), 43.3° (400), 53.6° (422), 57.8° (511), and 62.9° (440), which are related to the crystal phase of Fe_3_O_4_ nanoparticles.^11^ These results confirmed that Fe_3_O_4_ MNPs were successfully synthesized and did not any changes during synthesis of Fe_3_O_4_@boehmite NPs. These results are in agreement with the standard XRD pattern of Fe_3_O_4_ MNPs. Also, the boehmite phase in Fe_3_O_4_@boehmite NPs was characterized by the peak positions at 14.9 (020), 28.5 (120), 38.7 (031), 45.7 (131), 49.4 (051), 51.9 (200), 55.8 (151), 60.1 (080), 65.2 (231), 66.6 (002), 68.6 (171), and 72.1 (251) in the XRD pattern.^27^ These results confirmed that BNPS did not any changes after modification of Fe_3_O_4_ MNPs.

**Fig. 7:**
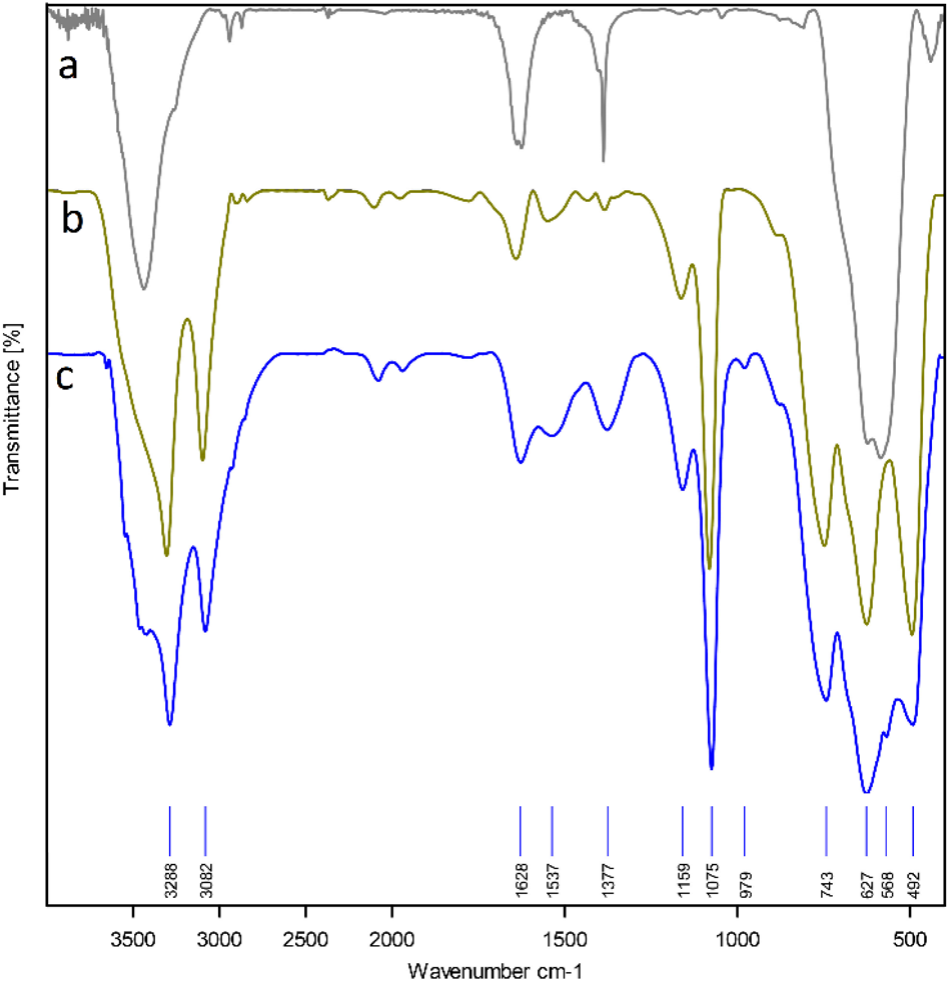
FT-IR spectra of Fe_3_O_4_ NPs (a), boehmite NPs (b) and Fe_3_O_4_@boehmite NPs (c).

The FT-IR spectrum of Fe_3_O_4_ NPs, boehmite NPs and Fe_3_O_4_@boehmite NPs are shown in Fig. 7. Two bands at region 443 and 587 cm^−1^ in the FT-IR spectrum of the Fe_3_O_4_ NPs (Fig. 7(a)) are correspond to the vibrations of the Fe–O bonds,^18,42^ which these bands are present in the FT-IR spectrum of Fe_3_O_4_@boehmite NPs (Fig. 7(c)). The peaks at (490, 621 and 744 cm^−1^) in the FT-IR spectrum of the boehmite NPs (Fig. 7(b)) related to the Al–O bonds vibrations,^27,44^ which these bands are also present in the FT-IR spectrum of Fe_3_O_4_@boehmite NPs (Fig. 7(c)). Hydrogen bands of OH⋯OH and nitrate impurity vibration were indicated by the several bands at (1158 and 1077 cm^−1^) and (1637 cm^−1^) respectively in FT-IR spectrum of the boehmite NPs (Fig. 7(b)).^42,43,45^

The surface of Fe_3_O_4_@boehmite NPs has a large number of hydroxyl groups which the stretching vibration of them appeared above 3000 cm^−1^ in the FT-IR spectrum.^17^ Also, the vibrations of their hydrogen bands of OH⋯OH are presented at 1159 and 1075 cm^−1^.^44,45^ The several bands at 492, 627 and 743 cm^−1^ are corresponds to the vibration of the Al–O bonds.^27,46^ The characteristic the nitrate impurity were emerged at 1628 cm^−1^.^44,47^ The peaks which shown in region 452 and 568 cm^−1^ are related to the vibrations of the Fe–O bond of Fe_3_O_4_ NPs^18^ that overlap with the vibrations of the Al–O bonds.

**Fig. 8:**
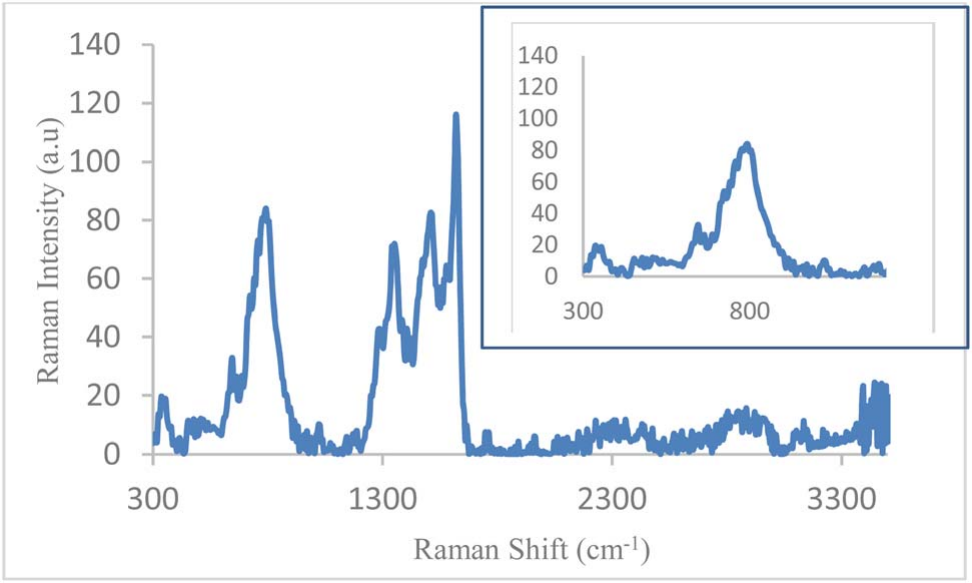
Raman spectrum Fe_3_O_4_@boehmite NPs.

The Raman spectrum of Fe_3_O_4_@boehmite NPs is shown in Fig. 8. The several bands at 312, 348, 513, 545, 663, 798, 1027, 1297, 1362, 1478, 1520, 1578, 1624, 1762, 2820, 2942 and 3150 cm^−1^ were observed in the Raman spectrum of Fe_3_O_4_@boehmite NPs. According to authentic literature, Fe_3_O_4_ NPs characterized by several peaks on 311, 540 and 665 cm^−1^ in Raman spectroscopy.^48^ Therefore, the Raman spectrum shows that Fe_3_O_4_@boehmite NPs contains Fe_3_O_4_ NPs, not Fe_2_O_3_ NPs. Also, based on another previous literature,^49,50^ Fe_3_O_4_ nanoparticles characterized by several peaks on 305, 513, 534 and 660 cm^−1^ in Raman spectroscopy. These peaks are observed in Raman spectrum of Fe_3_O_4_@boehmite NPs which are indexed to Fe_3_O_4_ NPs in Fe_3_O_4_@boehmite NPs. Meanwhile, based on same literature,^49,50^ γ-Fe_2_O_3_ nanoparticles characterized by several peaks on 350, 500 and 700 cm^−1^ in Raman spectroscopy and α-Fe_2_O_3_ nanoparticles characterized by several peaks on 221, 244, 292, 406, 497 and 611 cm^−1^ in Raman spectroscopy. These peaks were not observed in the Raman spectrum of Fe_3_O_4_@boehmite NPs. Therefore, Fe_3_O_4_@boehmite NPs only includes Fe_3_O_4_ NPs.

**Fig. 9:**
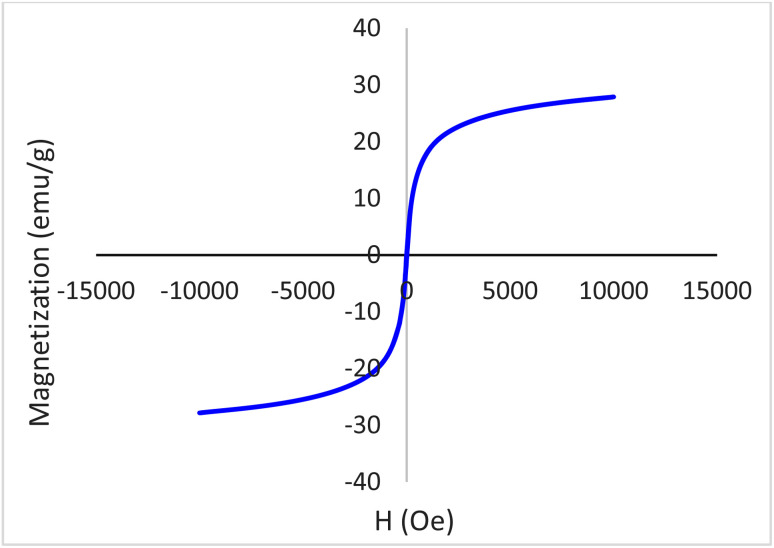
Magnetization curves for Fe_3_O_4_@boehmite NPs.

The magnetic property of Fe_3_O_4_@boehmite NPs was studied by VSM technique using LBKFB device from “Magnetic Kavir Kashan”. The VSM curve of Fe_3_O_4_@boehmite NPs is shown in Fig. 9. Magnetic properties of Fe_3_O_4_@boehmite NPs was found to be 27.5 emu g^−1^. As expected, Fe_3_O_4_@boehmite NPs showed the lower magnetic value in comparison to Fe_3_O_4_ NPs (which is about 74.09 emu g^−1^^12^) due to the coating of Fe_3_O_4_ nanoparticles by boehmite layers.

**Scheme 1:**
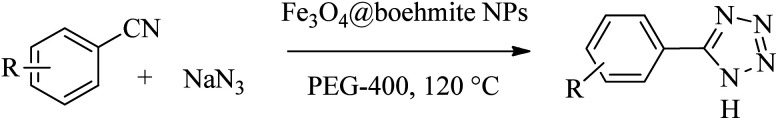
Synthesis of 5-substituted 1*H*-tetrazoles in the presence of Fe_3_O_4_@boehmite NPs.

After characterization of Fe_3_O_4_@boehmite NPs, its catalytic application was studied in the synthesis of 5-substituted 1*H*-tetrazole derivatives (Scheme 1).

Firstly, the [3+2] cycloaddition of NaN_3_ with benzonitrile in the presence of Fe_3_O_4_@boehmite NPs was selected for optimizing reaction conditions (Table 1). The effect of solvent, amount of NaN_3_ and temperature was studied in the presence of various catalytic amount of Fe_3_O_4_@boehmite NPs. As shown, the [3+2] cycloaddition of NaN_3_ with benzonitrile was tested in the presence of (30, 20, 15 and 10) mg of Fe_3_O_4_@boehmite NPs catalyst (Table 1, entries 2–5), which 15 mg of Fe_3_O_4_@boehmite NPs is optimal amount of this catalyst for completing of the reaction in acceptable time (Table 1, entry 4). Reducing the amount of catalyst from 15 mg to 10 mg significantly increases in the reaction time and reduces in the product yield (Table 1, entry 5). While increasing the amount of catalyst has insignificant effect on reaction time or yield (Table 1, entry 3). Then, the optimization conditions were continued in various solvents (Table 1, entries 6–9) in the presence of 15 mg (optimal amount) of Fe_3_O_4_@boehmite NPs catalyst. Among various solvents, the best results were obtained when PEG was used as solvent (Table 1, entry 4). Also in continuation of our studying the effect of temperature and sodium azide content was examined, which the best result was observed at 120 °C using 1.5 mmol sodium azide (Table 1, entry 4).

**Table 1:** Optimization reaction conditions for [3+2] cycloaddition of NaN_3_ with benzonitrile in the presence of Fe_3_O_4_@boehmite NPs catalyst

Entry	Catalyst (mg)	Solvent	NaN_3_ (mmol)	Temperature (°C)	Time (min)	Yield (%)^a^
1	—	PEG	1.5	120	600	—
2	30	PEG	1.5	120	210	95
3	20	PEG	1.5	120	220	93
4	15	PEG	1.5	120	240	97
5	10	PEG	1.5	120	600	88
6	15	PEG	1.7	120	230	90
7	15	H_2_O	1.5	120	240	25
8	15	DMSO	1.5	120	240	68
9	15	DMF	1.5	120	240	73
10	15	PEG	1.5	100	240	60

a Isolated yield.

After optimization of reaction conditions, the catalytic application of Fe_3_O_4_@boehmite NPs was extended to aromatic (Table 2, entries 1–12) and aliphatic (Table 2, entries 13–15) nitrile derivatives. In this regard, aromatic nitriles; including, an electron-donating or electron-withdrawing groups on aromatic ring were investigated for the synthesis of corresponding tetrazole deraivatives. As shown in Table 2, all products were obtained in acceptable times and excellent yields which indicated the excellent efficiency of Fe_3_O_4_@boehmite NPs. Also, *ortho*-, *meta*- and *para*-substituted benzonitriles were successfully investigated. More additions, aliphatic nitriles were investigated and corresponding tetrazoles were obtained in excellent yields.

**Table 2:** Synthesis of tetrazoles in the presence of Fe_3_O_4_@boehmite NPs

Entry	Nitrile	Product	Time (min)	Yield^a^ (%)
1	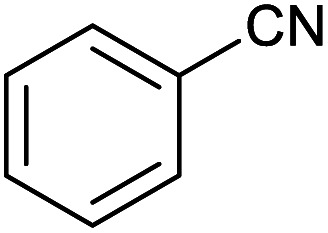	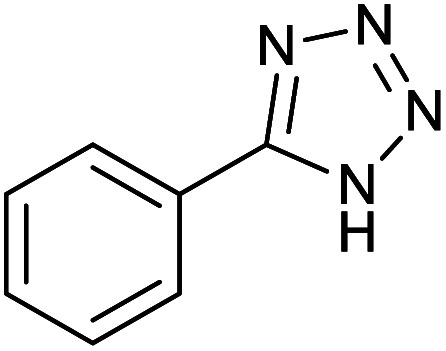	240	97
2	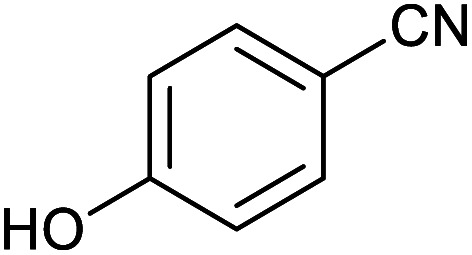	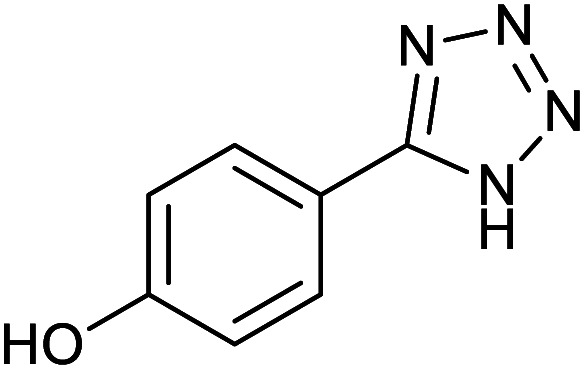	335	95
3	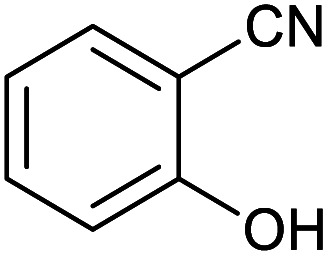	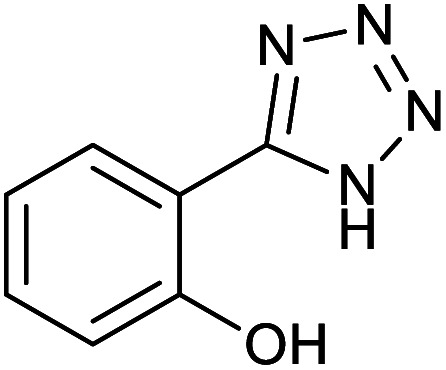	300	96
4	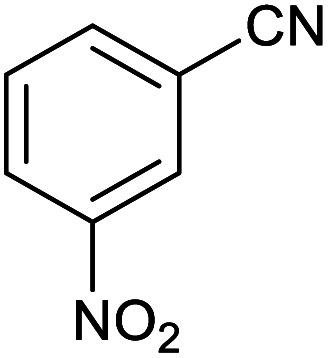	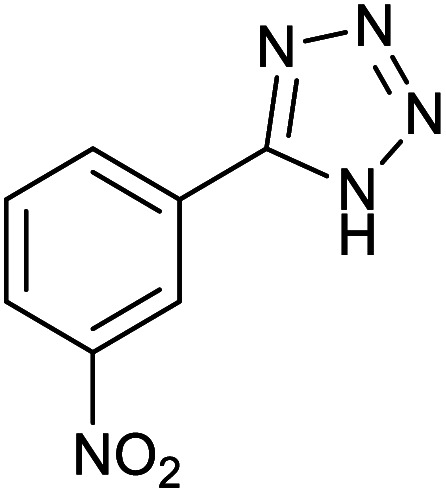	360	93
5	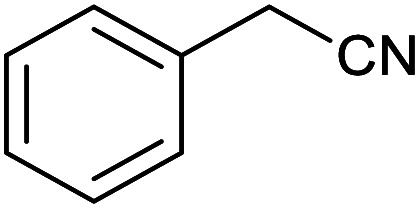	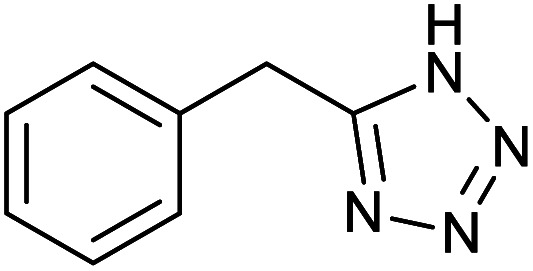	260	91
6	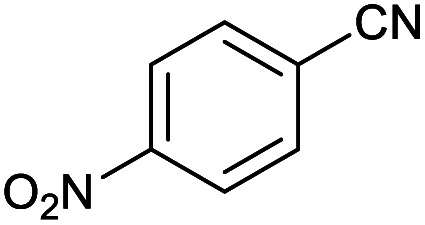	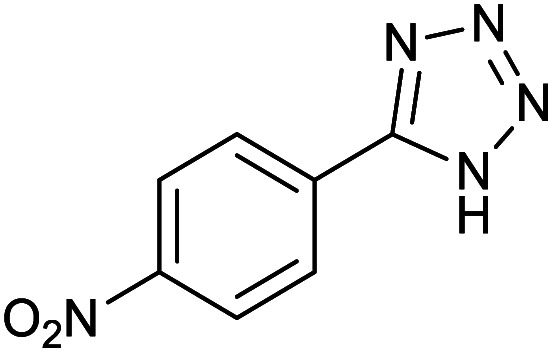	28 h	90
7	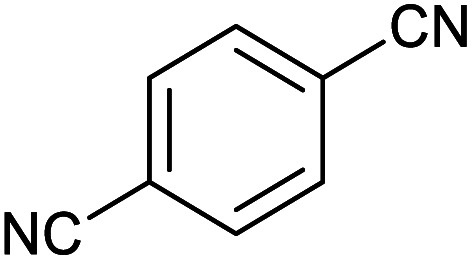	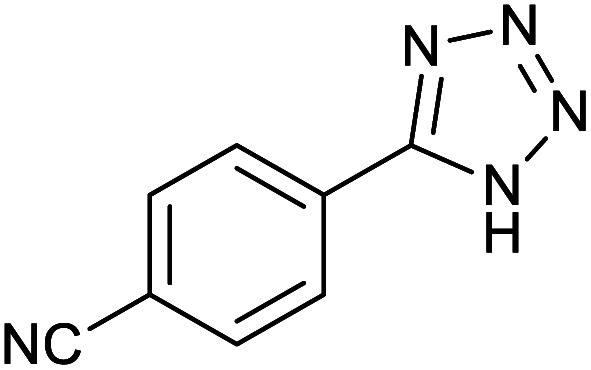	24 h	92
8	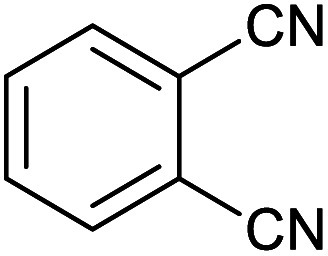	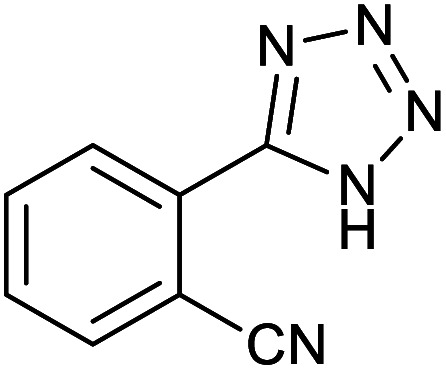	375	94
9	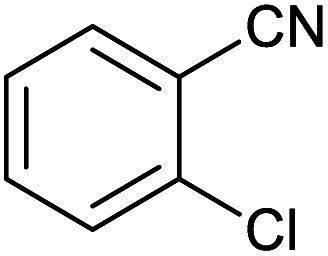	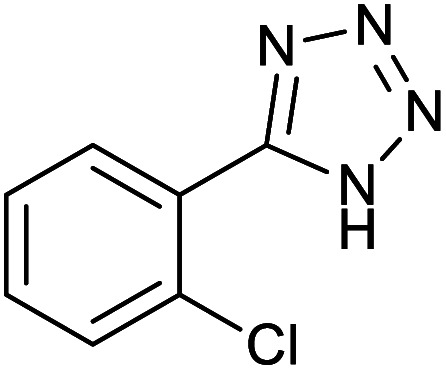	610	92
10	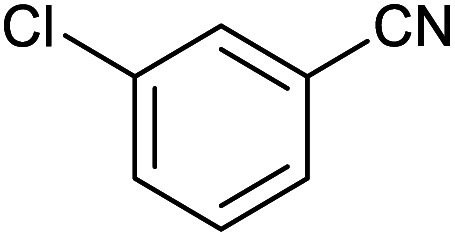	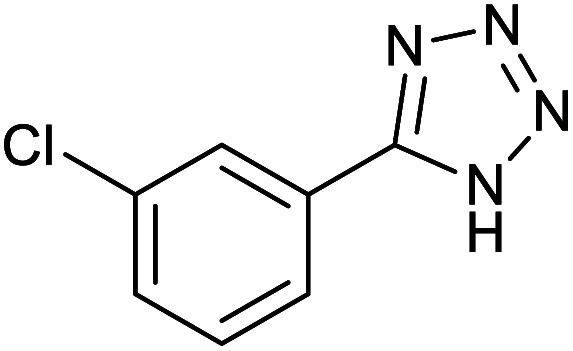	12.5 h	90
11	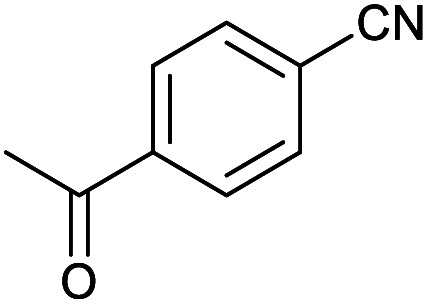	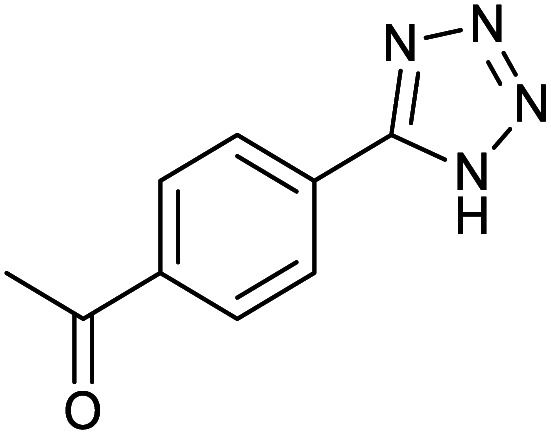	37 h	85
12	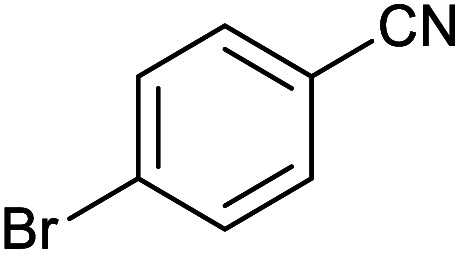	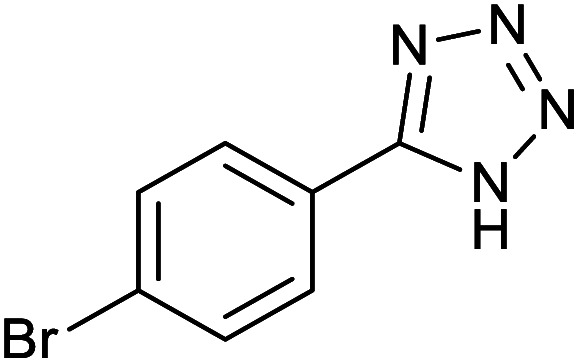	25	95
13	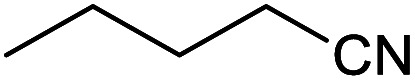	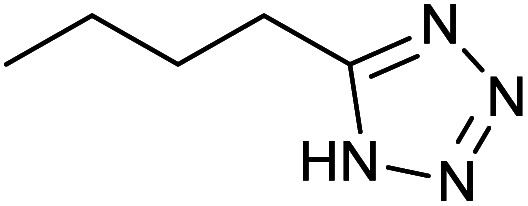	12 h	87
14	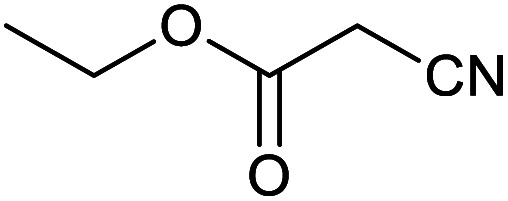	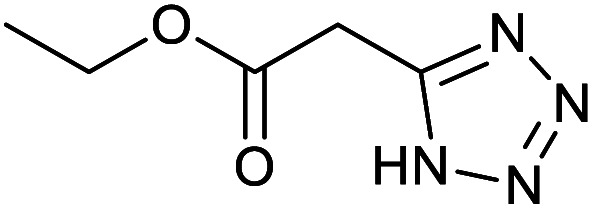	105	93
15	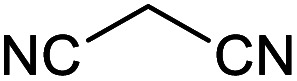	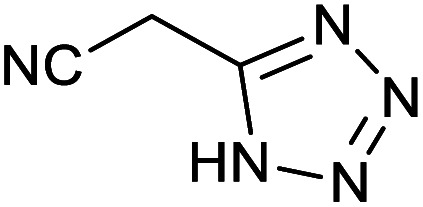	65	85
16	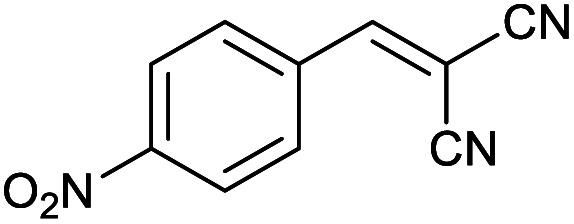	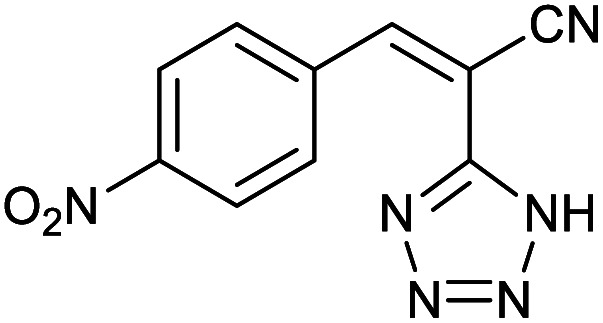	135	95
17	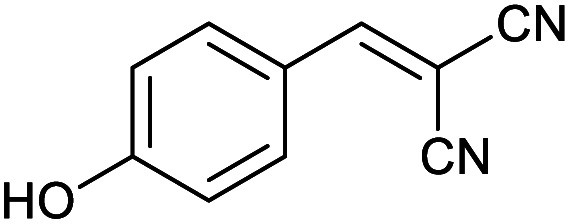	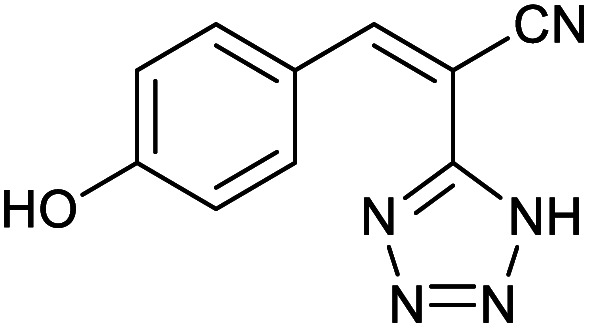	200	93

a Isolated yield.

**Scheme 2:**
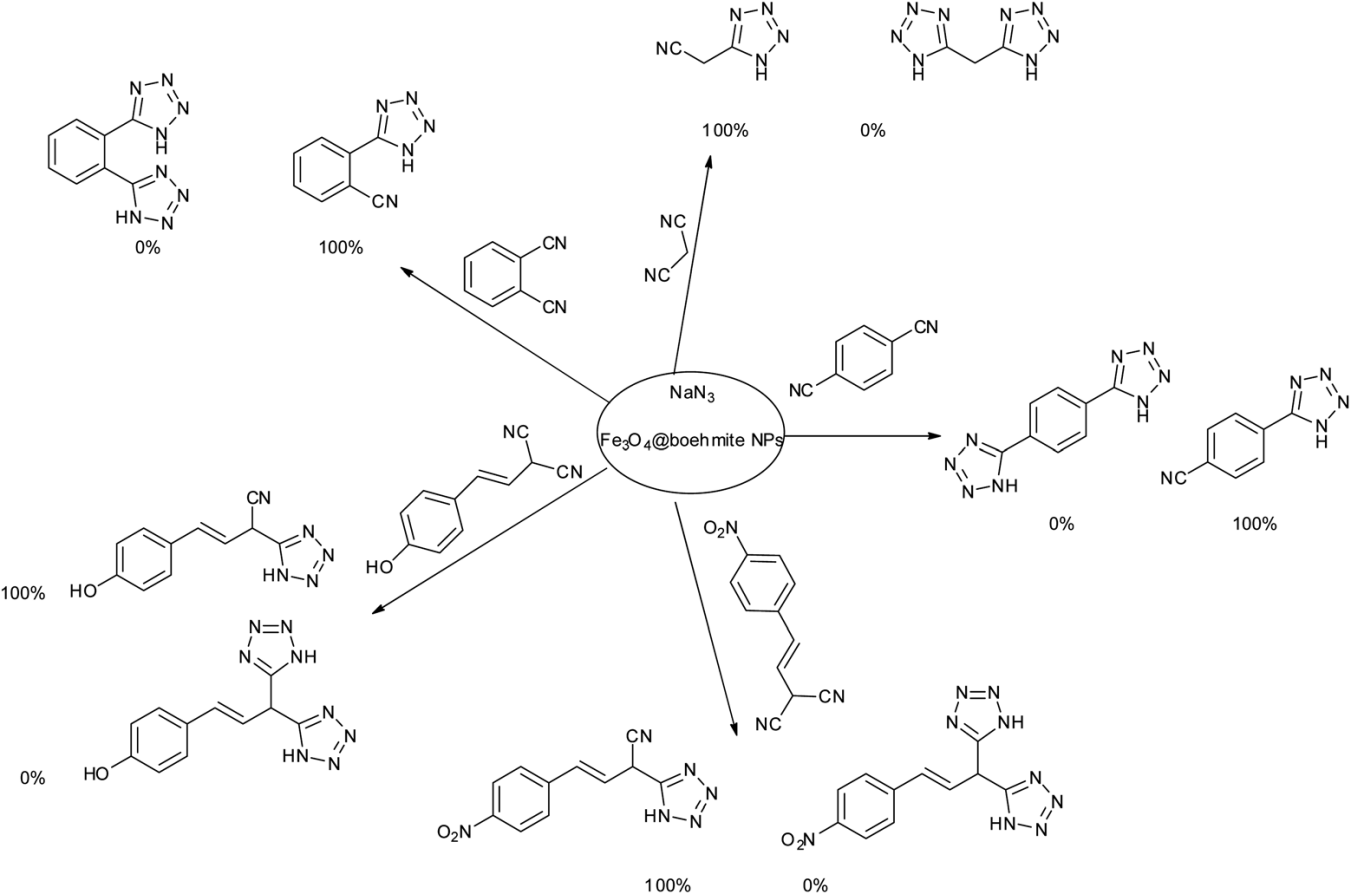
Homoselectivity of Fe_3_O_4_@boehmite NPs catalyst in the [3+2] cycloaddition of NaN_3_ with dicyano substituted derivatives.

Homoselectivity is an interesting property which indicate in the quite the same functional groups that only one of them take part in the reaction.^51,52^ For example, malononitrile which has two identical cyano groups, was investigated for the synthesis of corresponding tetrazoles in the presence of Fe_3_O_4_@boehmite NPs catalyst (Table 2, entry 15). Interestingly, this catalyst shown a good selectivity in the synthesis of tetrazoles and only one of identical cyano groups in malononitrile was reacted with sodium azide and another cyano groups was remained without any change (Scheme 2).

Also, benzylidenemalononitrile derivatives bearing electron-withdrawing (Table 2, entry 16) and electron–donor (Table 2, entry 17) functional groups were investigated for the synthesis of tetrazoles in the presence of Fe_3_O_4_@boehmite NPs catalyst. These substrate have two cyano-functional groups with same position in their structure which afforded the mono [3+2] cycloaddition with sodium azide in the presence of Fe_3_O_4_@boehmite NPs catalyst. Phthalonitrile and terphthalonitrile (Table 2, entries 7 and 8) were also investigated and the products were obtained in high yield and excellent homoselectivity (Scheme 2). Therefore, these results were indicated the high efficiency of Fe_3_O_4_@boehmite NPs catalyst in the synthesis of the wide range of tetrazoles.

**Fig. 10:**
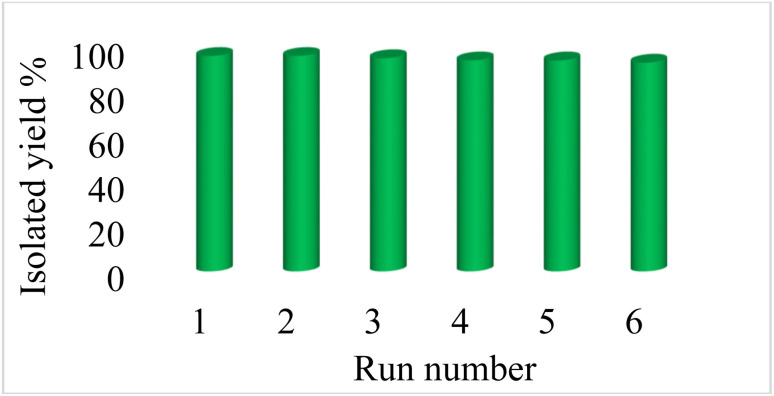
Recyclability of Fe_3_O_4_@boehmite NPs catalyst in the [3+2] cycloaddition of NaN_3_ with benzonitrile.

Reusability of catalysts is one of the principles of green chemistry in the design of chemical processes, which is the most important advantage of heterogeneous catalysts over homogeneous catalysts. Therefore, the reusability of Fe_3_O_4_@boehmite NPs catalyst was studied in the [3+2] cycloaddition of NaN_3_ with benzonitrile (Fig. 10). In this studying, in the end of each reaction, Fe_3_O_4_@boehmite NPs catalyst was recovered using an external magnet and washed with water and ethyl acetate. Subsequently the recovered catalyst was charged by same reactants for next run. As shown in Fig. 10, Fe_3_O_4_@boehmite NPs catalyst reused up to 6 times.

In order to studying the iron leaching from Fe_3_O_4_@boehmite NPs catalyst in the reaction mixture, the [3+2] cycloaddition of NaN_3_ with benzonitrile was repeated under optimized conditions. In the end of the reaction, the catalyst was isolated by external magnet and the amount of probably leached iron in the reaction solution was calculated by AAS analysis, which a significant amount of iron was not detected in the reaction solution. Therefore, Fe_3_O_4_@boehmite NPs are formed by strong bonds between boehmite and Fe_3_O_4_ NPs.

The efficiency and practically of Fe_3_O_4_@boehmite NPs catalyst was compared to previously catalysts in the synthesis of 5-phenyl-1*H*-tetrazole from the [3+2] cycloaddition of NaN_3_ with benzonitrile (Table 3). As shown the higher yield of the product was obtained in the presence of Fe_3_O_4_@boehmite NPs catalyst than other catalysts. Also, the reaction time in the presence of Fe_3_O_4_@boehmite NPs catalyst is shorter than other catalysts. Fe_3_O_4_@boehmite NPs catalyst has the advantages of both MNPs and BNPs and it can be reused for several times. While purification of the products and reusing of the homogeneous catalysts are often difficult, costly and time consuming. The synthesis of tetrazole derivatives were out come in PEG as green solvents in the presence of Fe_3_O_4_@boehmite NPs catalyst, while some previously procedures used organic solvents.

**Table 3:** Comparison of Fe_3_O_4_@boehmite NPs catalyst in the [3+2] cycloaddition of NaN_3_ with benzonitrile

Entry	Catalyst	Time (h)	Yield (%)	Ref.
1	Cu-TBA@biochar	7	98	26
2	CoY zeolite	14	90	53
3	Cu–Zn alloy nanopowder	10	95	54
4	B(C_6_F_5_)_3_	8	94	55
5	Fe_3_O_4_@SiO_2_/Salen Cu(ii)	7	90	56
6	Fe_3_O_4_/ZnS HNSs	24	81.1	57
7	Mesoporous ZnS	36	86	58
8	Cu(OAc)_2_	12	98	59
9	CuFe_2_O_4_	12	82	60
10	Nano ZnO/Co_3_O_4_	12	90	61
11	Cu(ii)-adenine-MCM-41	5	92	62
12	Pd-isatin-boehmite	8	94	63
13	Fe_3_O_4_@boehmite NPs	4	97	This work

## 4 Conclusions

In conclusion, magnetic boehmite nanoparticles was synthesized as an efficient and reusable heterogeneous catalyst for the homoselective synthesis of 5-substituted 1*H*-tetrazoles. This catalyst is composed of boehmite nanoparticles and Fe_3_O_4_ nanoparticles, therefore it has advantages of both boehmite nanoparticles (such as high surface area and stability) and Fe_3_O_4_ (such as easy separation by an external magnet) systems. Magnetic boehmite nanoparticles can be recovered and reused up to 6 times without any significant loss of its activity. Magnetic boehmite nanoparticles was characterized by TEM, SEM, EDS, WDX, ICP, FT-IR, Raman, XRD and VSM techniques.

## Conflicts of interest

There are no conflicts to declare.

## Supplementary Material

RA-012-D2RA04759D-s001
