# Supplementary material for: Investigation of Fe3O4@boehmite NPs as efficient and magnetically recoverable nanocatalyst in the homoselective synthesis of tetrazoles
Source: RSC Adv. 2022 Nov 22;12(52):33459–68. doi: 10.1039/d2ra04759d (PMC9680009; doi:10.1039/d2ra04759d)

Electronic Supplementary Material (ESI) for RSC Advances.
This journal is © The Royal Society of Chemistry 2022

Supplementary Data

Investigation of Fe_3O_4@boehmite NPs as efficient and magnetically recoverable nanocatalyst in the homoselective synthesis of tetrazoles

Parisa Moradi*

Department of Chemistry, Faculty of Science, Ilam University, P. O. Box69315516, Ilam, Iran. ; E-mail address: parisam28@yahoo.com

Abstract:

Magnetic boehmite nanoparticles (Fe_3O_4@boehmite NPs) were synthesized from a hybrid of boehmite and Fe_3O_4 nanoparticles. At first, boehmite nanoparticles (aluminum oxide hydroxide) were prepared *via* a simple procedure in water using commercially available materials such as sodium hydroxide and aluminum nitrate. Then, these nanoparticles were magnetized using Fe_3O_4 NPs in a basic solution of $FeCl_2.4H_2O$ and $FeCl_3.6H_2O$. Fe_3O_4@boehmite NPs have advantages of both boehmite nanoparticles and Fe_3O_4 magnetic materials. Magnetic boehmite nanoparticles have been characterized by various techniques such as TEM, SEM, EDS, WDX, ICP, FT-IR, Raman, XRD and VSM. SEM and TEM images confirmed that particles size are less than 50 nm in diameter with a cubic orthorhombic structure. Then, Fe_3O_4@boehmite NPs were applied as a homoselective, highly efficient, cheep, biocompatibility, heterogeneous and magnetically recoverable nanocatalyst in the synthesis of 5-substituted 1H-tetrazole derivatives. Fe_3O_4@boehmite NPs can be recycled for several runs in the synthesis of tetrazoles. Also, all tetrazoles were isolated in high yields, which reveals high activity of Fe_3O_4@boehmite NPs in the synthesis of tetrazole derivatives. Fe_3O_4@boehmite NPs shows a good homoselectivity in synthesis of 5-substituted 1H-tetrazole derivatives.

* Address correspondence to P. Moradi, Department of Chemistry, Ilam University, P.O. Box 69315516, Ilam, Iran; Tel/Fax: +98 841 2227022; E-mail address: parisam28@yahoo.com or p.moradi@ilam.ac.ir

^{1}H NMR spectral data

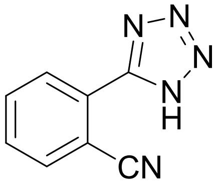

2-(1H-tetrazol-5-yl)benzonitrile

^{1}H NMR (400 MHz, $CDCl_3$): δ_H= 8.11-8.06 (t, *J*= 8 Hz, 2H), 7.96-7.90 (t, *J*= 12 Hz, 1H), 7.81-7.75 (t, *J*= 20 Hz, 1H)ppm.

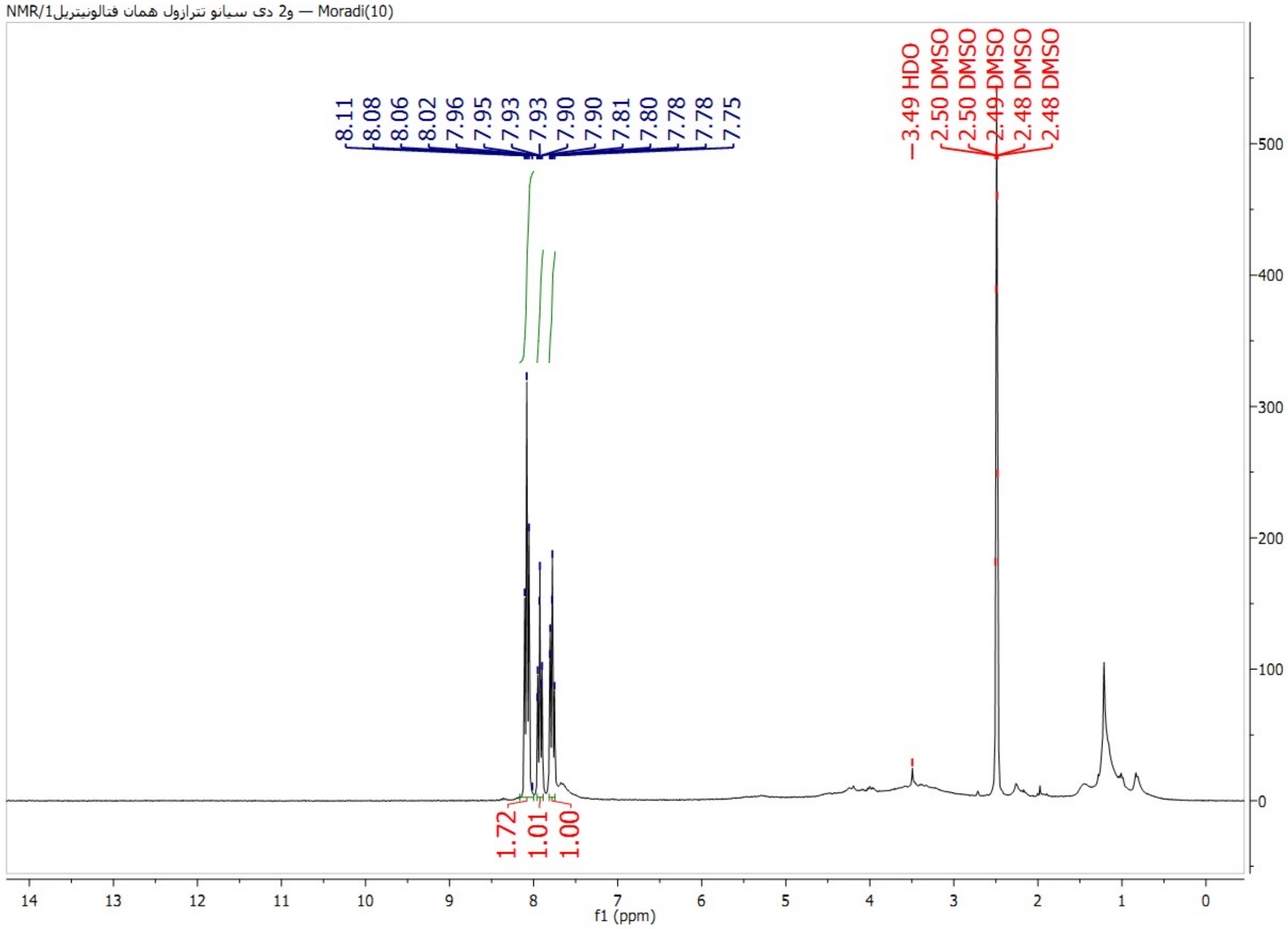

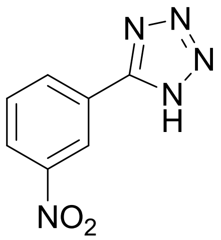

5-(3-nitrophenyl)-1H-tetrazole

^{1}H NMR (400 MHz, $CDCl_3$): δ_H= 14.96 (br, 1H), 8.84-8.83 (t, *J*= 4 Hz, 1H), 8.49-8.39 (m, 2H), 7.93-7.88 (t, *J*= 8 Hz, 1H) ppm.

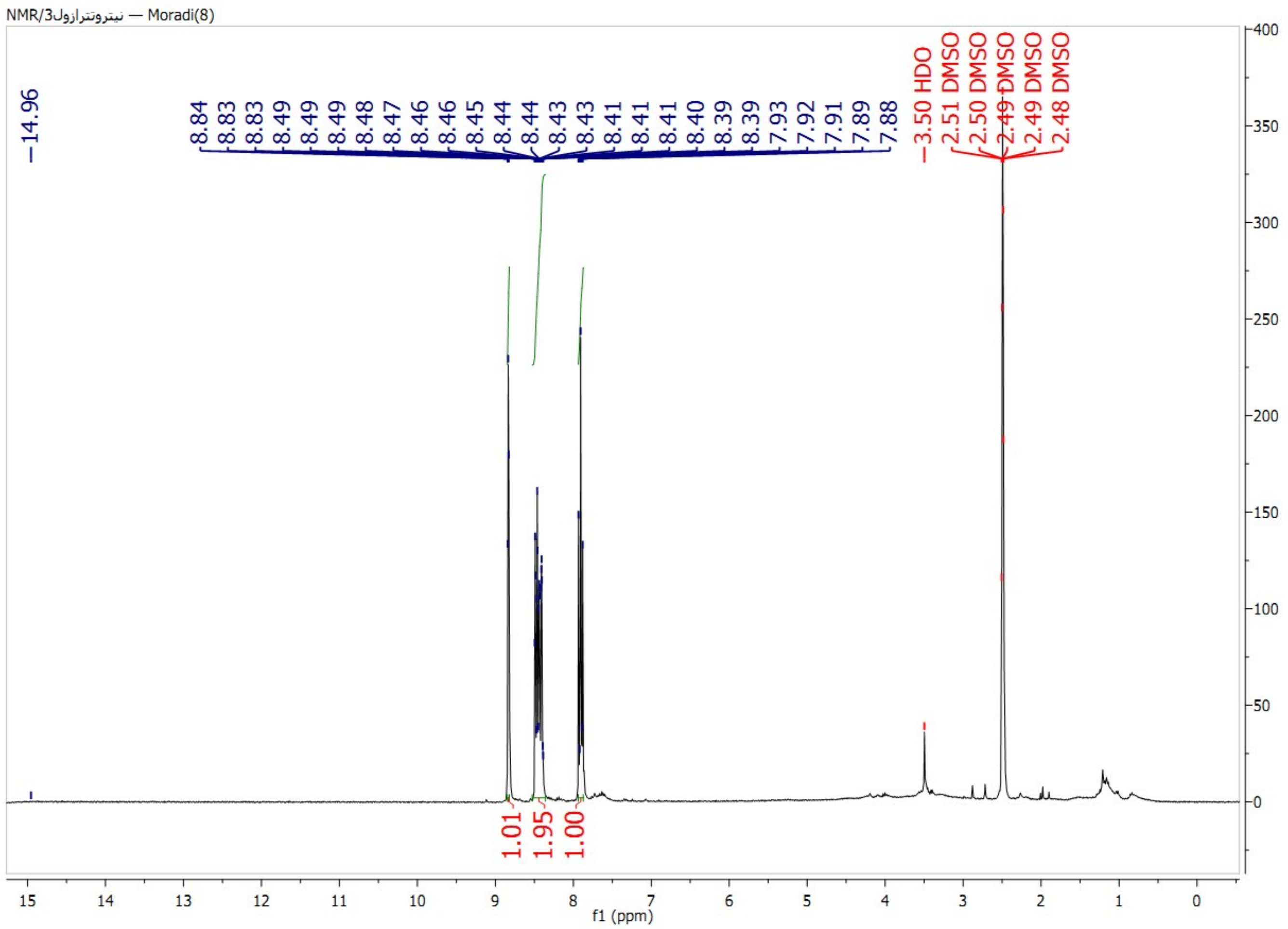

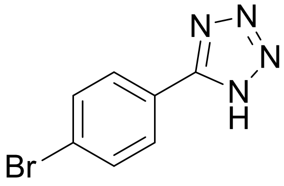

5-(4-bromophenyl)-1H-tetrazole

^{1}H NMR (400 MHz, $CDCl_3$): δ_H= 7.98-7.96 (d, *J*= 8 Hz, 2H), 7.84-7.82 (d, *J*= 8 Hz, 2H) ppm.

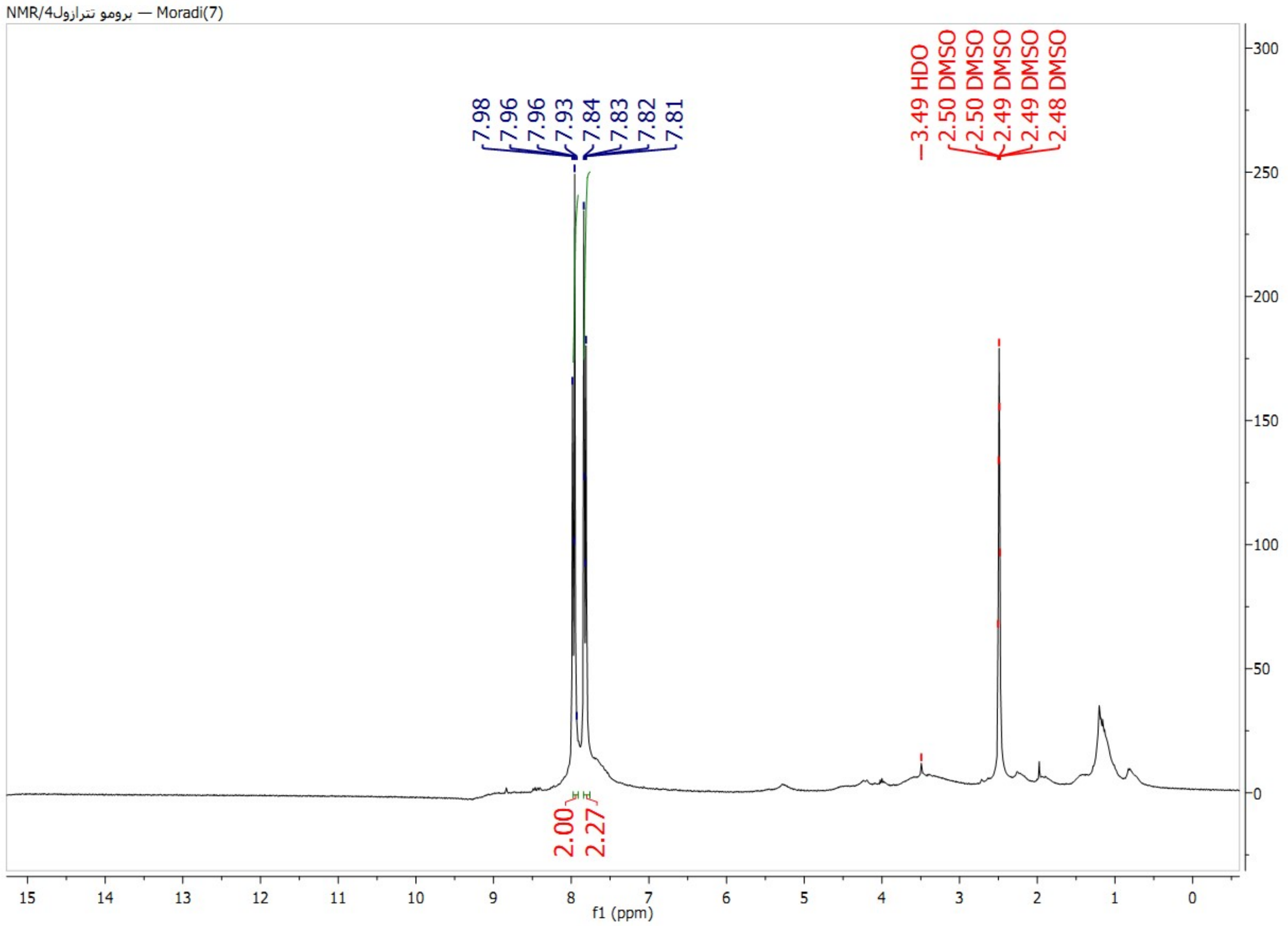

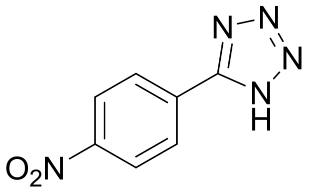

5-(4-nitrophenyl)-1H-tetrazole

^{1}H NMR (400 MHz, $CDCl_3$): δ_H= 14.76 (br, 1H), 8.45-8.41 (d, *J*= 12 Hz, 2H), 8.30-8.27 (d, *J*= 12 Hz, 2H) ppm.

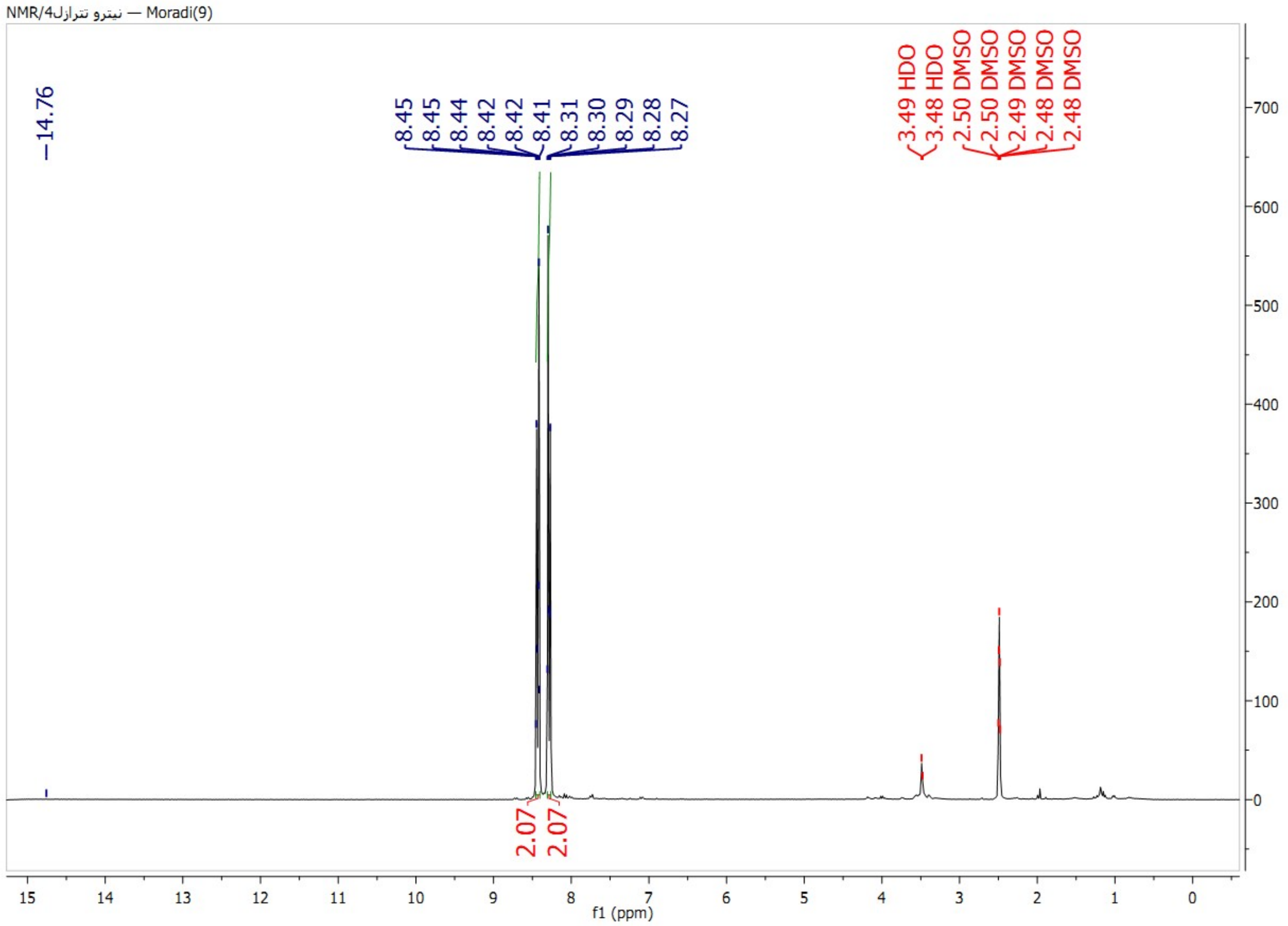

Supplement: RA-012-D2RA04759D-s001 [file RA-012-D2RA04759D-s001.pdf]
